# MicroRNA Signatures of the Developing Primate Fovea

**DOI:** 10.3389/fcell.2021.654385

**Published:** 2021-04-08

**Authors:** Elizabeth S. Fishman, Mikaela Louie, Adam M. Miltner, Simranjeet K. Cheema, Joanna Wong, Nicholas M. Schlaeger, Ala Moshiri, Sergi Simó, Alice F. Tarantal, Anna La Torre

**Affiliations:** ^1^Department of Cell Biology and Human Anatomy, University of California, Davis, Davis, CA, United States; ^2^Department of Ophthalmology, University of California, Davis, Davis, CA, United States; ^3^Department of Pediatrics, University of California, Davis, Davis, CA, United States; ^4^California National Primate Research Center, University of California, Davis, Davis, CA, United States

**Keywords:** microRNAs, retinal development, fovea, miR-342-5p, miR-15b, rhesus monkey

## Abstract

Rod and cone photoreceptors differ in their shape, photopigment expression, synaptic connection patterns, light sensitivity, and distribution across the retina. Although rods greatly outnumber cones, human vision is mostly dependent on cone photoreceptors since cones are essential for our sharp visual acuity and color discrimination. In humans and other primates, the *fovea centralis* (fovea), a specialized region of the central retina, contains the highest density of cones. Despite the vast importance of the fovea for human vision, the molecular mechanisms guiding the development of this region are largely unknown. MicroRNAs (miRNAs) are small post-transcriptional regulators known to orchestrate developmental transitions and cell fate specification in the retina. Here, we have characterized the transcriptional landscape of the developing rhesus monkey retina. Our data indicates that non-human primate fovea development is significantly accelerated compared to the equivalent retinal region at the other side of the optic nerve head, as described previously. Notably, we also identify several miRNAs differentially expressed in the presumptive fovea, including miR-15b-5p, miR-342-5p, miR-30b-5p, miR-103-3p, miR-93-5p as well as the miRNA cluster miR-183/-96/-182. Interestingly, miR-342-5p is enriched in the nasal primate retina and in the peripheral developing mouse retina, while miR-15b is enriched in the temporal primate retina and increases over time in the mouse retina in a central-to-periphery gradient. Together our data constitutes the first characterization of the developing rhesus monkey retinal miRNome and provides novel datasets to attain a more comprehensive understanding of foveal development.

## Introduction

Sight is often considered our most fundamental sense to perceive and navigate the world and, as a result, vision loss has a devastating impact on everyday life. Visual perception begins when photons of light enter the eye and are absorbed by the photoreceptors, the light-sensitive cells of the retina. There are two classes of photoreceptors named rods and cones because of their distinctive morphologies. While both populations contribute to the information transmitted to the visual centers of the brain by the optic nerve, these two cell types serve different purposes: rods are highly sensitive to light and provide relatively coarse, colorless images, while cones require considerably brighter light and are responsible for our sharp chromatic vision and spatial acuity (Arshavsky and Burns, 2012).

All photoreceptors are localized in the outer nuclear layer of the retina and are organized in a mosaic pattern that varies in different organisms to fit their environments and behaviors (Raymond et al., 1995; Bruhn and Cepko, 1996; Szel et al., 1996; Fadool, 2003; Viets et al., 2016). In most mammals, rods outnumber cones by orders of magnitude; in the mouse retina, rods constitute 97.2% and cones are 2.8% of all photoreceptors [38:1 rod to cone ratio, (Carter-Dawson and LaVail, 1979; Jeon et al., 1998)], while the human retina contains an average of 92 million rods and 4.6 million cones [20:1 rod to cone ratio, (Curcio et al., 1987)]. In humans and other primates, most of the cones are confined to a small region of the central temporal retina called the *macula lutea* (macula), where the cone concentration is about 200-fold higher than the most eccentric retinal regions (Curcio et al., 1987, 1990; Hendrickson et al., 2012; Hendrickson, 2016). At the center of the macula, a small indentation marks the location of the *fovea centralis* (fovea) corresponding with the center of the visual field. At the foveal pit, all photoreceptors are densely packed cones and there are virtually no rods (O’Brien et al., 2004; Springer and Hendrickson, 2005; Dubis et al., 2012; Provis et al., 2013). Despite the overall predominance of rod photoreceptors, primates have evolved to primarily utilize cone pathways, and most of our useful photopic vision depends on the cones in the fovea such that a 2-millimeter lesion in this area will result in legal blindness.

Age-related macular degeneration (AMD), one of the most prevalent types of photoreceptor degeneration, affects millions of people worldwide, and causes irreversible vision loss from the selective degeneration of the photoreceptors of the fovea (Wong et al., 2014). It has been estimated that AMD affects up to 25% of the United States population over the age of 80 (Friedman et al., 2004), illustrating the urgent need for novel treatments to restore the cones of the fovea. Efforts to develop therapies aimed at cone replacement will inevitably require preclinical studies using non-human primates, but our understanding of primate retinogenesis is still incomplete. Similarly, despite the fundamental importance of the fovea for human vision, the molecular mechanisms that guide the development of this region as well as the pathways that regulate the higher ratios of cone production remain largely unresolved.

During retinal development, different classes of retinal populations are consecutively added in a well-known sequence that is conserved in all vertebrates (Sidman, 1961; Young, 1985; Cepko et al., 1996; Livesey and Cepko, 2001): Retinal ganglion cells (RGCs), cone photoreceptors, and horizontal cells are the first cell populations to be born, followed by amacrine cells and rod photoreceptors, and finally, bipolar cells and Müller glia are born last. Classic lineage-tracing studies showed that retinal progenitor cells are multipotent such that one single type of progenitor cell has the ability to differentiate into multiple postmitotic cell types. Evidence from heterochronic transplants (McConnell, 1985; Watanabe and Raff, 1990; Belliveau et al., 2000; Rapaport et al., 2001), in which neural progenitors were transplanted into an environment of a different age and, more recently, single-cell transcriptomics (Clark et al., 2019; Lu et al., 2020; Sridhar et al., 2020) has revealed that (1) retinal progenitors are intrinsically restricted, and (2) retinal progenitors pass through waves of competence to acquire and lose the ability to make specific cell types at different developmental stages.

MicroRNAs (miRNAs) are small RNA molecules known to regulate several aspects of development. To date, over 2,000 miRNAs have been recorded in miRbase (miRbase.org) (Kozomara and Griffiths-Jones, 2014) and both computational and experimental analyses indicate that most protein-coding genes are regulated by one or more miRNAs (Baek et al., 2008; Selbach et al., 2008). The essential roles of miRNAs in cell fate acquisition and central nervous system (CNS) patterning are well established. miRNAs are known to regulate neural progenitor competence *in vivo* (Georgi and Reh, 2011; La Torre et al., 2013; Saurat et al., 2013; Shu et al., 2019; Wohl et al., 2019) and *in vitro* (Andersson et al., 2010; Patterson et al., 2014), and some miRNAs have been associated with the production of specific cell types (Bian et al., 2013; Nowakowski et al., 2013; Patterson et al., 2014; Wohl and Reh, 2016).

Given the vast importance of miRNAs as developmental regulators, we have sought to characterize the miRNome of the early developing non-human primate retina, specifically the rhesus monkey (*Macaca mulatta*), an Old World non-human primate. We have generated transcriptomic profiles of rhesus retinas at three developmental time points, spanning the major stages of development, and we have used miRNA-sequencing technologies to identify miRNAs differentially expressed in the presumptive fovea (temporal posterior side of the retina) compared to its equivalent region at the other side of the optic nerve head (nasal posterior) at early stages of retinal development. In addition, we have chosen miRNAs with significant differential expression between retinal regions and we have validated their expression using *in situ* hybridization in mouse and human samples. Together, our data provides invaluable resources for studies aimed at understanding the role of miRNAs in retinal development as well as datasets to broaden our knowledge of foveal development.

## Results

### Transcriptomic Characterization of the Developing Rhesus Monkey Retina

Total RNA was obtained from retinal punches (approximately 2.5 mm in diameter) from the prospective fovea (temporal side) and the equivalent region at the other side of the optic nerve head (nasal side), from three different time points spanning the three trimesters [50 days gestational age (late first trimester), 90 days (second trimester), and 150 days (third trimester); term 165 ± 10 days]. Rhesus monkey trimesters are divided by 55-day increments (0–55, 56–110, and 111–165 days) (Tarantal, 2005). We performed Next Generation Sequencing (NGS) analyses (50 days: 6 samples, 3 temporal, and 3 nasal, 90 days and 150 days: 2 samples each, 1 temporal, and 1 nasal for each ontogenic stage). After the pre-processing pipeline and quality controls, more than 89% of the reads were aligned with the rhesus monkey genome (reference genome: Mmul_1; annotation reference: Ensembl_75) for each sample. On average, 74.9 million reads were obtained from each sample, and genome mapping was on average 90%.

**FIGURE 1:**
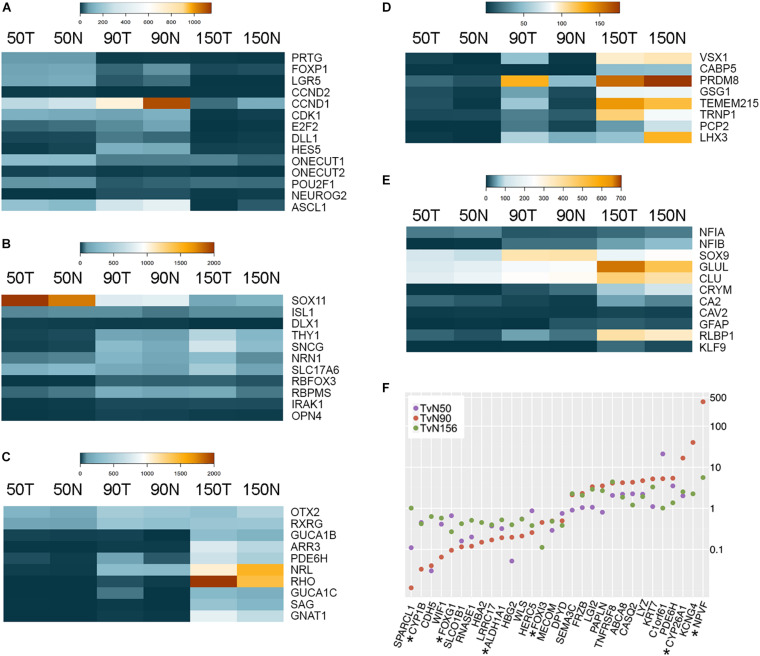
RNA-sequencing of *Macaca mulatta* retinas. **(A–E)** Heatmaps showing expression of cell-specific markers during *Macaca mulatta* retinal development. Comparisons between Temporal (T) and Nasal (N) data is shown at 50, 90, and 150 days gestational age. **(A)** Retinal Progenitor Cell markers. **(B)** Retinal Ganglion Cell markers. **(C)** Photoreceptor cell markers. **(D)** Bipolar cell markers. **(E)** Muller Glia cell markers. All data is shown as CPM (counts per million). **(F)** Scatter plot of genes showing differences between temporal and nasal expression. TvN: Temporal vs Nasal. Stars indicate genes previously identified as macula-enriched.

We used the expression of cell type-enriched genes as a read-out of the timing of retinal histogenesis (Figures 1A–E and Supplementary Table1). As expected, by 50 days gestational age, several well-known progenitor genes are highly expressed (e.g., PRTG, FOXP1) but not all progenitor genes reach the highest expression point at these early stages and several progenitor genes such as bHLH transcription factors (e.g., ASCL1, NEUROG2) and genes associated with active proliferation (e.g., CCND1, CDK4, E2F1, and E2F2) do not peak until 90 days gestational age (Figure 1A and Supplementary Figure 1A). Previous reports have identified clear transcriptional differences between early and late retinal progenitor competence states in mouse and human retinas (Clark et al., 2019; Lu et al., 2020; Sridhar et al., 2020), including a progressive increase in Notch signaling. The activation of the Notch pathway maintains cells in a proliferative state ensuring that a subset of progenitors remains for the consecutive waves of neurogenesis (Perron and Harris, 2000; Louvi and Artavanis-Tsakonas, 2006). Notch also regulates fate decisions through the regulation of neurogenic genes (Kageyama et al., 2008; Maurer et al., 2014). Correspondingly, many genes involved in the Notch signaling pathway show their highest levels of expression at 90 days gestational age in our screening (Supplementary Figure 1B), with NOTCH1, NOTCH3, DLL1, DLL3, and HES5 peaking at this time.

Similarly, genes known to be expressed in both mouse and human RGCs exhibit specific expression at different time points (Figure 1B). For example, several transcription factors such as ISL1 and SOX11 are highly expressed at early stages of development corresponding with their expression in other species (Mu et al., 2008; Pan et al., 2008; Jiang et al., 2013), while genes associated with RGC synaptic maturation (e.g., NRN1 or SNCG) increase over developmental time and peak in the third trimester. Interestingly, many photoreceptor-specific (Brzezinski and Reh, 2015) and bipolar cell-specific markers (Park et al., 2017) are detected first in the temporal samples before the nasal samples starting from 50 days gestational age (Figures 1C,D). Accordingly, by this time, the temporal samples exhibit higher levels of cone genes (e.g., PDE6H, 3.5-fold enrichment; Figure1C) and early bipolar genes (e.g., VSX1 shows a 28-fold enrichment, GSG1 shows a 3.1-fold enrichment, and TMEM215 shows an 8.3-fold enrichment; Figure 1D). These differences between temporal and nasal regions are more prominent in the second trimester (90 days gestational age), reflecting a vast developmental acceleration in the presumptive fovea. Thus, by this time, the expression of GUCA1B is 14.5-fold higher in the temporal samples, PDE6H shows an enrichment of 5.8-fold (photoreceptor markers), and the bipolar markers VSX1, CABP5, PRDM8, GSG1, TMEM215 are enriched 39.8-, 2.6-, 2.8-, 4.5- and 10.3-fold, respectively. Correspondingly, many Müller glia-specific genes are up-regulated over developmental time, including NFIX, GLUL, CA2, and RLBP1 (Figure 1E).

In addition to the cell-specific markers, other genes also exhibit transcriptional differences between the temporal and nasal regions of the developing rhesus eye (Figure 1F and Supplementary Tables 1, 2). Notably, many of these genes have been previously shown to be differentially expressed in the developing macula or the high-acuity area of other species. For example, FOXG1 is a transcription factor exclusively expressed in the nasal portion of the retina in fish, chicken, mouse, and human (Shintani et al., 2004; Zhao et al., 2009; Fotaki et al., 2013; Hernandez-Bejarano et al., 2015; Hoshino et al., 2017; Smith et al., 2017). Hoshino and collaborators demonstrated that CYP1B1 is enriched in the periphery of the human fetal retina (Hoshino et al., 2017). Notably, CYP26A1 is higher in the temporal retina at all the ages analyzed and ALDH1A1 is enriched in the nasal retina. CYP26A1 and ALDH1A1 are negative and positive regulators of retinoic acid (RA) levels, respectively, and downregulation in RA signaling correlates with the development of a rod-free area in the avian retina (da Silva and Cepko, 2017). CYP26A1 and NPVF have also been previously identified as developing macula markers in human samples by different reports (Hoshino et al., 2017; Lu et al., 2020). Additionally, our analyses also identify novel genes such as CROC4 (C1orf61), CASQ2, SPARCL1, and WIF1 as genes presenting strong differential expression signatures between the presumptive fovea and the nasal side at different gestational time points (Figure 1F). Collectively, these results show that the rhesus monkey presumptive fovea is developmentally advanced relative to the opposite nasal region, confirming the utility of these data as a tool to analyze differences between temporal and nasal expression.

### miRNA-Sequencing and Differential Expression Profiles Between Temporal and Nasal Fetal Rhesus Monkey Retinas

**FIGURE 2:**
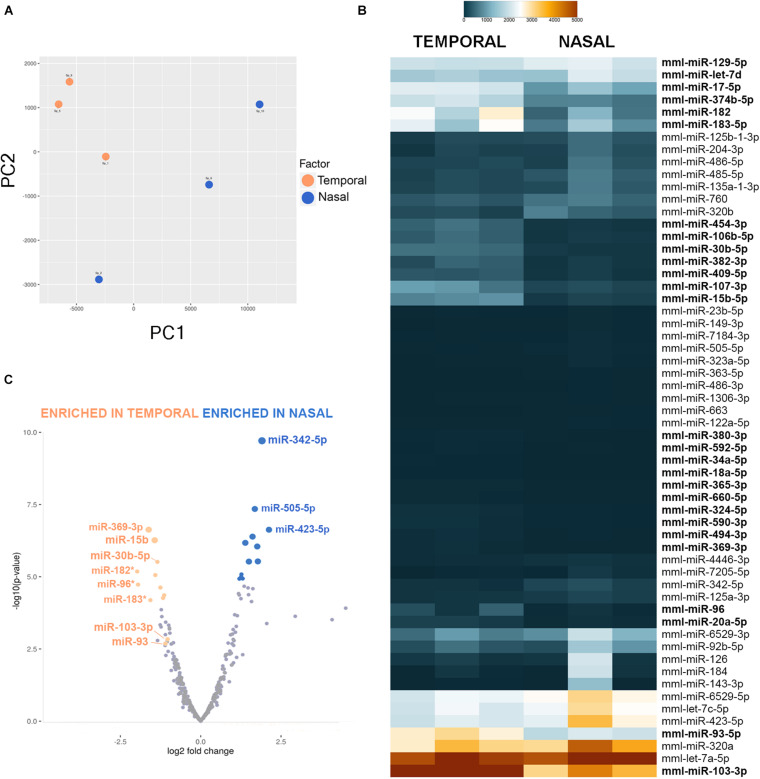
MiRNA-sequencing of *Macaca mulatta* retinas. **(A)** Two-dimensional Principal Component Analysis was used to visualize sample variance between six retinal tissue samples collected at 50 days gestational age. **(B)** Heatmap shows different miRNA expression between temporal and nasal samples. Only miRNAs with a False Discovery Rate (FDR) value of < 0.012 between nasal and temporal samples are shown. miRNAs enriched in the temporal samples are shown in bold. **(C)** Volcano plot of miRNA plotted on fold change (log2, x-axis) and *P* value [−log10(*p*-value), y-axis] shows difference in expression between nasal and temporal retina. Stars indicate the photoreceptor enriched miR-183/96/182 cluster.

miRNA libraries were obtained from retinal punches from the temporal side of the retina (presumptive fovea) and the nasal side of the optic nerve head as described above, at 50 days gestational age (*n* = 3 samples for each anatomical region, six samples total). After NGS profiling, an average of 29.9 million reads were obtained per sample, and the data was mapped to miRBase (release 20) and normalized. Principal Component Analysis (PCA) was performed by including the top 50 microRNAs that varied the most across all samples using normalized reads. As shown in Figure 2A, the foveal/temporal samples form a relatively robust cluster indicating that the biological differences between these samples are pronounced despite the nasal samples exhibiting larger intra-group variability.

Importantly, our experiments indicate that several miRNAs are differentially expressed in the different regions of the developing primate retina (Figures 2B,C). For example, miR-183, miR-96, and miR-182 are significantly enriched in the developing fovea (3.3-fold, 5.21-fold, and 5.11-fold, respectively). miR-183/-96/-182 are co-expressed together as a single primary transcript, are highly expressed in developing photoreceptors in mouse and zebrafish, and play essential roles in photoreceptor development and maintenance (Zhu et al., 2011; Xiang et al., 2017; Fogerty et al., 2019). Since the temporal region of the developing primate eye exhibits both a developmental acceleration and higher percentage of photoreceptors, it is not surprising that this family is expressed at greater levels in the temporal samples. Additionally, several other miRNAs are significantly enriched in the presumptive fovea compared to the nasal samples, including miR-369-3p (4.86-fold enrichment with a *p*-value of 2.45E-7), miR-15b-5p (3.85-fold enrichment with *p*-value of 5.6E-7), miR-30b-5p (3.96-fold enrichment with a *p*-value of 8.99E-6), miR-103-3p (2.69-fold enrichment with a *p*-value of 0.0009), and miR-93-5p (2.57-fold enrichment with a *p*-value of 0.0019). However, the expression levels of miR-369-3p are fairly low (Figure 2B). In contrast, miR-342-5p is significantly enriched in the nasal site (2.71-fold with a *p*-value of 1.96E-10).

Interestingly, miRNAs belonging to the same families often show similar expression profiles: all members of the miR-17/-20/-93/-106/-519 family are enriched over 2-fold in the temporal samples, miR-15b and miR-16 are enriched 3.85-fold and 2.65-fold, respectively, and miR-130a/-130b/-454/-301 are also all expressed at higher levels in the temporal region of the eye (Supplementary Table 3). This suggests that these miRNA families are frequently regulated as a whole, perhaps at the primary transcript stage.

Previous studies have indicated that miRNAs coordinately regulate protein levels and thus, miRNAs that target the same complexes are often co-expressed (Sass et al., 2011). We have used MIENTURNET [MicroRNA ENrichment TURned NETwork, (Licursi et al., 2019)] to gain insight into the possible miRNA networks in the different regions of the primate retina (Supplementary Figure 2). The network analyses of some of the highest expressed miRNAs for each region suggest possible differences in cell cycle regulation as several cell cycle genes including CCND1, CDKN1A, TP53, and CCNE1 are potentially regulated by differentially expressed miRNAs (Supplementary Tables 4, 5). Similarly, FOXG1 is potentially targeted by miR-30b-5p and miR-103-3p while NFIA and NFIB, two transcription factors involved in fate specification in the retina (Clark et al., 2019), are potentially targeted by miR-30b-5p, miR-103-3p, and miR-93 (Supplementary Figure 2 and Supplementary Table 5). Additionally, several genes involved in the NOTCH (DLL1), WNT (WNT3A, AXIN2), FGF (FGF4, FGF18), and RA (RORB, RORA) pathways are also targeted by the temporal miRNA network.

**FIGURE 3:**
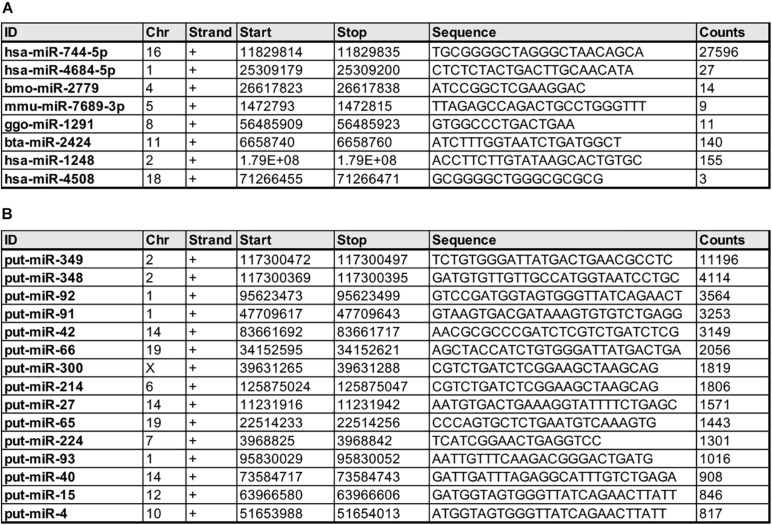
Novel *Macaca mulatta* miRNAs at 50 days gestational age. **(A)** miRNAs previously discovered in other species but unknown to be expressed in *Macaca mulatta* prior to this study. **(B)** Putative novel miRNAs based on counts and secondary precursor hairpin structure.

Notably, our analyses also reveal several miRNAs that were not previously annotated in the *Macaca mulatta* database but known in other species (Figure 3A) as well as putative novel miRNAs (Figure 3B), based on counts and putative secondary precursor hairpin structures identified using the miRPara software (Wu et al., 2011).

### miRNA Expression in the Developing Mouse Retina

The miRNAs identified in our screening could be differentially expressed in the developing fovea for various reasons; for example, since the fovea is developmentally accelerated, temporally-regulated miRNAs are expected to increase first in the temporal side of the retina. Similarly, miRNAs enriched in cell populations found in higher percentages in the fovea (e.g., cones) could also exhibit higher expression levels in the temporal samples. Finally, the progenitors of the fovea could possess unique properties and miRNA signatures.

**FIGURE 4:**
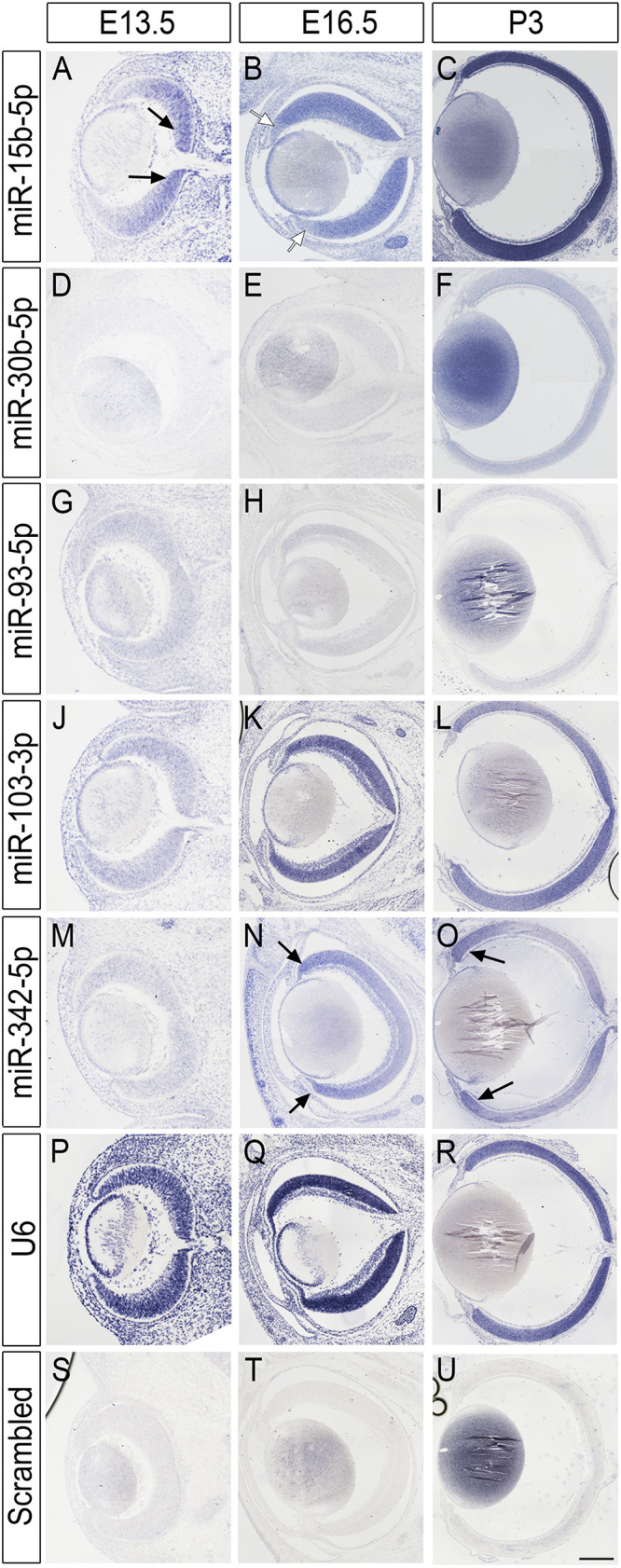
miRNA *in situ* hybridization in the mouse retina. **(A–U)** miRNA expression in the murine retina at E13.5, E16.5, and P3. Rows are labeled with the miRNA probe used, including U6 positive control and scrambled negative control. Each column shows a different developmental time point. Black arrows indicate increased expression in central **(A)** or peripheral **(N,O)** retina regions. White arrows indicate lower expression **(B)**. Scale bars: 40 microns for panels **(A,D,G,J,M,P,S)** (first column), 100 microns for panels **(B,E,H,K,N,Q,T)** (second column), and 500 microns in panels **(C,F,I,L,O,R,U)** (last column).

Since miRNA-seq technologies do not offer cellular resolution, and given the scarcity of primate samples, we first attempted to validate the developmental expression and cellular resolution of the top miRNA candidates using mouse tissue at three different time points: embryonic day 13.5 (E13.5), E16.5, and postnatal day 3 (P3) by *in situ* hybridization (ISH) (Figure 4). All the miRNAs tested show some level of expression in the murine samples and, in all cases, the expression detected was above the labeling threshold in negative controls (scrambled probe, Figures 4S–U). miR-15b, miR-30b, and miR-103-3p are up-regulated over the time points analyzed and show the highest levels of expression at P3 (Figures 4A–F,J–L). Interestingly, miR-15b exhibits a clear center-to-periphery pattern and it is first detected in the central retina at E13.5 (black arrows, Figure 4A and Supplementary Figure 3). By E16.5, most of the retina expresses miR-15b, but we found lower levels of expression in the peripheral tips (Figure 4B, white arrows) and the whole retina expresses high levels of miR-15b by P3. miR-30b is expressed throughout the thickness of the retina at P3 but we observed a moderate enrichment in the ganglion cell layer (GCL) and the basal part of the inner nuclear layer, suggesting higher expression in amacrine cells and possibly RGCs (Figure 4F and Supplementary 3). Interestingly, miR-93 expression is missing from the apical side of the retina at P3, suggesting that this miRNA may be expressed at lower levels in developing murine photoreceptors (Figure 4I and Supplementary 3). Finally, miR-342-5p shows higher expression levels in the peripheral retina from E16.5 onward (Figure 4N and Supplementary Figure 3), and this pattern of expression is maintained by P3 (Figure 4O, black arrows). In contrast, positive control experiments (U6 probe, Figures 4P–R) show neither regional differences nor changes in expression coordinated with the stage of development.

### Expression in the Developing Human Retina

**FIGURE 5:**
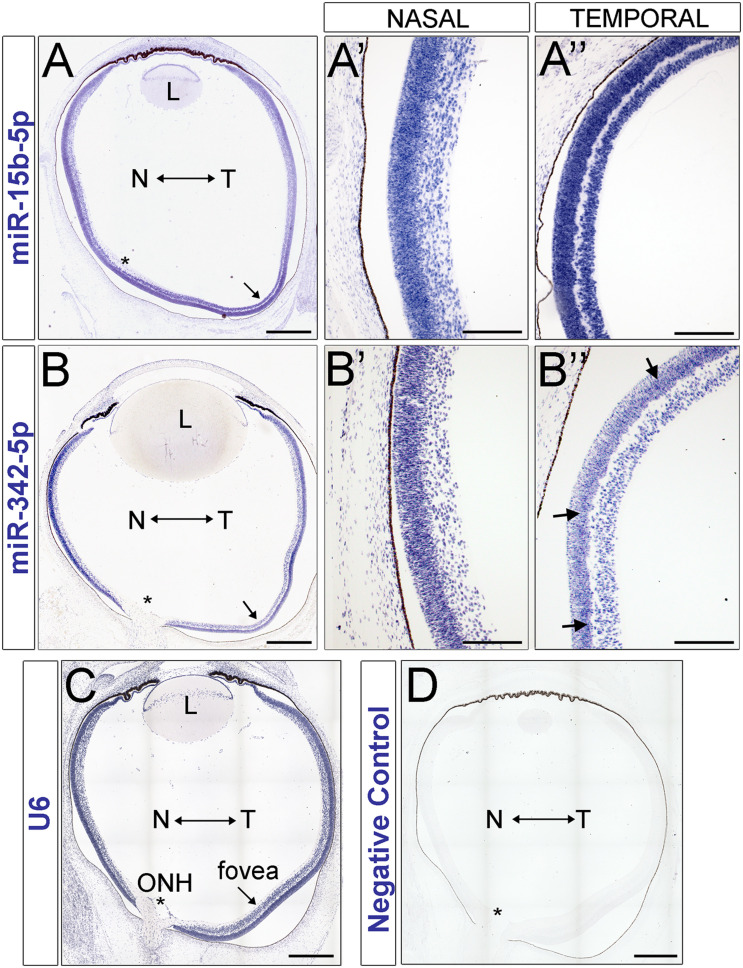
miRNA *in situ* hybridization in the human retina. **(A–D)** miRNA expression in the human fetal retina at 70–82 days gestation. miR-15b **(A–A″)** and miR-342-5p **(B–B″)** expression in the developing human retina. **(A,B)** tiled montage of the whole eye. **(A′,B′)** inset of nasal retina. **(A″,B″)** inset of temporal retina at the presumptive fovea. **(C)** U6 positive control. **(D)** ISH negative control. L, Lens; N, Nasal; T, Temporal; ONH, Optic Nerve Head. Asterisks indicate the location of the optic nerve head and the arrows indicate the presumptive foveal region. Scale bars: 500 microns in A, B, C and D, 200 microns in panels **(A′,A″,B′,B″)**.

The experiments using murine samples indicate that several of the miRNAs identified in our miRNA-seq screening are developmentally regulated and that miR-15b and miR-342-5p also show compartmentalized expression with central-to-peripheral differences. To further assess whether these expression patterns are conserved in primates and relevant to human biology, we used human fetal retina tissue to test miRNA expression of our top candidates (Figure 5 and Supplementary Figure 6). Since the rhesus samples were obtained at 50 days gestational age (30% gestation), we collected human fetal samples at gestational ages estimated to be between 77–83 gestational days (28–31% of gestation, Supplementary Figure 4). In order to obtain additional data on the developmental stage of the samples assessed, we performed immunohistochemistry using known markers and Hematoxylin and Eosin staining (Supplementary Figure 5). At the stage analyzed, there are PCNA+ retinal progenitors in all the quadrants of the retina, but the thickness of the neuroblastic layer where the retinal progenitors reside is thinner on the temporal side (NbL, Supplementary Figures 5A–C′). Similarly, we detected fewer PH3+ mitotic cells on the temporal site of the retina compared to the nasal side (arrows, Supplementary Figures 5B–C′,F), indicating that more progenitors have already exited the cell cycle in this region. Correspondingly, we also detected increased numbers of OTX2+ photoreceptors on the temporal side of the eye (Supplementary Figures 5D–G′).

Remarkably, miR-15b is expressed at higher levels in the temporal side of the optic nerve head (Figures 5A–A″) and miR-342-5p shows higher labeling signals in the nasal side of the eye (Figures 5B–B″) as predicted by our miRNA-seq analyses (Figures 2B,C). Both miRNAs display stronger signal in the neuroblast layer compared to other regions of the retina, including the GCL and the most apical side of the retina where the developing photoreceptors reside (arrows in Figure 5B″ and Supplementary Figure 7). In contrast, we did not detect significant miR-93 or miR-30b differences between the temporal and nasal retina (Supplementary Figure 6). As shown previously, our U6 positive control is ubiquitously expressed (Figure 5C) and our negative controls (Figure 5D) show very low levels of non-specific labeling.

## Discussion

Our most advanced visual abilities such as reading and recognizing faces are dependent on the highly-specialized structure of the fovea. Unfortunately, the current understanding of retinal development is primarily based on mouse studies. Since the mouse retina does not contain a fovea and the rod-to-cone ratio in rodents resembles the most eccentric regions of the human retina, the molecular events that lead to the formation of the macula and the cone-dominated fovea remain largely unknown.

Previous studies have shown that the primate retina develops over many months and, in fact, the human fovea is not fully developed until 4 years of age (Hendrickson et al., 2012). Histological data using human and non-human primate samples has revealed that retinal development takes place in a dramatically compartmentalized manner such that two regions separated by a few millimeters may be at vastly different ontogenic stages (Yuodelis and Hendrickson, 1986; Xiao and Hendrickson, 2000; Hendrickson et al., 2008; Hendrickson, 2016). Moreover, primate retinal development does not progress in a central-to-peripheral gradient similar to mice but advances in a fovea-to-periphery manner. Consistent with this species-specific difference in patterning, the expression of S-Opsin and L/M-Opsin is first detected in the fovea (Cornish et al., 2004a,b). Recently, the first transcriptional profiling datasets of the developing human retina have been published using both human fetal tissue and stem cell organoids (Hoshino et al., 2017; Lu et al., 2020; Sridhar et al., 2020). However, obtaining human tissue at very early or late stages of development is challenging and these resources are subject to ethical and political issues (Ledford, 2017). In contrast, non-human primate models offer a unique opportunity to decipher some of the molecular mechanisms that dictate foveal development. The genus *Macaca*, probably the most extensively used non-human primate model (Roska and Sahel, 2018; Picaud et al., 2019), shares with humans susceptibility genes for AMD (Francis et al., 2008; Pahl et al., 2012; Yiu et al., 2017) and for other photoreceptor pathologies such as achromatopsia (Moshiri et al., 2019). Consequently, a comprehensive characterization of the specific mechanisms that regulate rhesus retinal development could facilitate the study of the pathophysiological events that lead to these diseases and enable the development of clinical approaches aimed at vision restoration.

Here, we provide the first spatio-temporal transcriptional datasets of the developing rhesus monkey retina obtained from temporal and nasal regions at three different gestational time points spanning all trimesters. The current study has limitations, including a modest sample size at some developmental time-points. Additionally, the incomplete annotation of the *Macaca mulatta* genome poses some challenges as the assembly contains many gaps, sequencing errors and misassembled scaffolds (Zhang et al., 2012; Norgren, 2013).

By analyzing the expression of cell-specific markers, our data offers insights into the timing of retinal histogenesis and indicates that by 50 days gestational age, the temporal side of the retina is already more developmentally advanced when compared to the nasal side (Figure 1). A model developed by Finlay (Finlay and Darlington, 1995), Clancy (Clancy et al., 2001), and Workman (Workman et al., 2013) and available at translatingtime.org explores the idea that timing of many neurodevelopmental events—such as the timing of retinal neurogenesis—is highly conserved among species and thus, can be predicted with high accuracy taking into account the growth rates for the different species. According to this model (Supplementary Figure 4), by 50 days gestational age (end of the first trimester), the rhesus monkey retina is at the peak of cone genesis and approximately at the onset of bipolar cell genesis. Assuming that neurogenesis for all the different cell types begins at the foveal region, these predictions appropriately fit our RNA-seq data. Indeed, by 50 days, we detect higher temporal expression of several bipolar genes, including GSG1 and TMEM215 (Figure 1D), two genes identified by the Brzezinski group as cone bipolar markers (Park et al., 2017). Our data also indicate that the maturation of photoreceptor cells follows a fovea-to-periphery gradient (Figure 1C), in agreement with histological evidence (Hendrickson and Zhang, 2019). Importantly, we also distinguish other genes differentially expressed in the presumptive developing fovea (Figure 1F), including genes previously identified in the human macula and in the avian high-acuity area as well as novel genes, such as the gene encoding for the calcium-binding binding protein Calsequestrin-2 and SPARCL1/Hevin. Future studies will shed light on the role of these genes in retinal development.

It has been proposed that the accelerated developmental timing of the fovea may be partially responsible for its unique cellular composition. During the sequence of retinal cell specification, cones are generated earlier than rods and thus, precocious cell cycle exit from the retinal precursor pool would result in increased representation of early cell types (e.g., cones). Comparison between diurnal (foveated) and nocturnal (afoveated) New World primates suggested that alterations in cell cycle kinetics could explain some of the differences between these models, including the higher production of cones in foveated species (Dyer et al., 2009). However, molecules associated with rod photoreceptor differentiation such as NRL and NR2E3 are never detected in the foveal region while other late cell types (e.g., bipolar cells and Müller glia) are present in the presumptive fovea before the cell movements that lead to pit formation (Yanni et al., 2012; Hoshino et al., 2017). Thus, it is feasible that the progenitors of the fovea possess unique characteristics that result in the stark difference in cell composition.

Prior studies have revealed that miRNAs are key regulators of the temporal changes that allow progenitors to produce different cell populations as development proceeds (Georgi and Reh, 2010; La Torre et al., 2013; Wohl et al., 2019). Similarly, we have also shown that miRNAs coordinate cell cycle kinetics (Fairchild et al., 2019). Given that the fovea exhibits both different cell composition and perhaps different cell cycle dynamics, we have characterized the miRNome of the early developing primate retina with the goal to pinpoint miRNAs differentially expressed in the progenitors of the fovea. We identified several miRNAs with different temporal and nasal expression levels. Among these, miRNA-183/96/182, a miRNA cluster highly expressed in photoreceptors and vital in maintaining cone photoreceptor outer segments (Busskamp et al., 2014; Zuzic et al., 2019) is significantly enriched in the temporal samples. Similarly, other miRNAs including miR-15b and miR-342-5p also showed significant differences in our datasets and we utilized ISH to further validate these differences using mouse and human fetal samples. According to our assessment, the human samples used in this study are in a developmental stage comparable to the rhesus monkey samples we used for the miRNA-seq (Supplementary Figure 4). Remarkably, miR-15b showed higher labeling in the temporal retina while miR-342-5p exhibited lower expression in the temporal side of the retina. Past studies in different models and contexts have revealed that miR-15b plays roles in cell cycle regulation and survival (Cimmino et al., 2005) while miR-342–5p acts downstream of Notch to regulate neural stem cell fate choices (Gao et al., 2017). This raises the possibility that one or both of these miRNAs may contribute to the molecular events that lead to the development of the central primate retina. Future studies aimed at the identification of the miRNA-mediated networks in conjunction with the existing human and primate expression datasets may shed light on the regulatory events that orchestrate the cytoarchitecture of the primate fovea.

## Materials and Methods

### Experimental Models and Subject Details

#### Rhesus Monkeys

All animal procedures conformed to the requirements of the Animal Welfare Act and protocols were approved prior to implementation by the Institutional Animal Care and Use Committee (IACUC) at the University of California at Davis. Normal, healthy adult female rhesus monkeys (*Macaca mulatta*) were bred and identified as pregnant using established methods (Tarantal, 2005). Pregnancy in the rhesus monkey is divided into trimesters by 55-day increments, with 0–55 days representing the first trimester, 56–110 days representing the second trimester, and 111–165 days gestational age the third trimester (term 165 ± 10 days). Normal embryonic/fetal growth and development were confirmed by ultrasound across gestation and until tissue collection (Tarantal, 2005). Dams were scheduled for hysterotomy (e.g., approximately 50, 90, or 150 days gestational age) for fetal tissue collection. Dams were returned to the breeding colony post-hysterotomy.

The fetal eyes were collected in cold PBS and the retinas were immediately dissected. With the cornea facing up, we made a small puncture in the center of the cornea with an 18 gauge needle. Using spring scissors (10 mm tip), we slowly cut the cornea from the puncture toward the corneo-scleral junction. We successively rotated the eye 90° and made three more cuts and we gently removed the lens. Then, using one of the cuts, we carefully inserted the lower blade of the scissors between the sclera/RPE and the retina and we cut all the way to the optic nerve head being careful not to damage the retina. We repeated using the other cuts at the corneo-scleral divide. Next, the sclera, RPE, and choroid were carefully removed with fine forceps (World Precision Instruments, Dumont tweezers 0.05 mm × 0.01 mm tips) to dissect the retina away from the rest of the tissues. We performed two cuts in the dorsal and ventral part of the retina to open its cup shape and the temporal and nasal samples were obtained using 2.5 mm biopsy punches (World Precision Instruments) at equidistant regions about 0.5 mm from the ONH. As the total size of the retina changes during development, the percentage of retina captured at the different stages varied in the different samples. At 50 days gestational age, the biopsy captured more than half of the retina from the ONH to the ora serrata, thus extending beyond the foveal anlage.

#### Mice

Pregnant CD-1 IGS females were obtained from Charles River and housed until embryos or neonates were at the proper developmental stage for dissection and fixation. All animals were used with approval from the University of California Davis IACUC. Dams were euthanized and embryos were dissected and fixed for ISH as described below.

#### Human Fetal Samples

Eyes (*n* = 6) were obtained from discarded de-identified human fetal tissue with permission of the University of California, Davis Institutional Review Board. The age for the human specimens was estimated by clinic intakes.

### RNA and miRNA Sequencing

#### Library Preparation and Next Generation Sequencing

Upon dissection, all the tissues were preserved in RNAlater (Thermo Fisher) at −80°C. Then, total RNA was obtained from all the samples using the Total RNA Purification plus micro kit (Cat #48500, Norgen), and we used an Agilent Bioanalyzer 2100 to evaluate the quality of the RNA obtained.

The sequencing experiments were conducted by Exiqon (Denmark). The library preparation was performed using Illumina TruSeq^®^ Stranded Total RNA (with Ribo-Zero Gold) preparation kit.

The starting material (1,000 ng) of total RNA was depleted of rRNAs using ribo-zero gold (to remove both cytoplasmic and mitochondrial rRNA) magnetic bead-based capture-probe system (Illumina Inc.). The remaining RNA (including mRNAs, lincRNAs and other RNA species) was subsequently purified (RNAcleanXP) and fragmented using enzymatic fragmentation. Then, first strand synthesis and second strand synthesis were performed, and the double stranded cDNA was purified (AMPure XP). The cDNA was end repaired, 3′ adenylated and Illumina sequencing adaptors ligated onto the fragments ends, and the library was purified (AMPure XP). The stranded libraries were amplified with PCR and purified (AMPure XP). The libraries size distribution was validated and quality inspected on a Bioanalyzer (high sensitivity DNA chip). High quality libraries were quantified using qPCR, the concentration normalized, and the samples pooled. The library pool(s) were re-quantified with qPCR and optimal concentration of the library pool used to generate the clusters on the surface of a flowcell before sequencing on a Nextseq500/High Output sequencing kit (51 cycles according to the manufacturer instructions (Illumina Inc.) using 50-bp single-end reads and 30 million reads.

### Sequence Analyses

Our data analysis pipeline is based on the Tuxedo software package, including Bowtie2 (v. 2.2.2), Tophat (v2.0.11), and Cufflinks (v2.2.1). CummeRbund was used for post-processing Cufflinks and Cuffdiff results. The heatmap.2 function contained within the ggplot2 R package was used to produce all heat maps. Transcriptomic heat maps were produced by selecting genes that represent specific retinal cell types based on established literature using normalized CPM values.

#### miRNA-Sequencing: Library Preparation and Next Generation Sequencing

For miRNA-sequencing, we used the same samples that were used for RNA-seq. A total of 500 ng of total RNA was converted into microRNA NGS libraries using NEBNEXT library generation kit (New England Biolabs Inc.) according to the manufacturer’s instructions. Each individual RNA sample had adaptors ligated to its 3′ and 5′ ends and converted into cDNA. Then the cDNA was pre-amplified with specific primers containing sample specific indexes. After 18 PCR cycles the libraries were purified on QiaQuick columns and the insert efficiency evaluated by a Bioanalyzer 2100 instrument on high sensitivity DNA chip (Agilent Inc.). The microRNA cDNA libraries were size fractionated on a LabChip XT (Caliper Inc.) and a band representing adaptors and 15–40 bp insert excised using the manufacturer’s instructions. Samples were then quantified using qPCR and concentration standards. Based on quality of the inserts and the concentration measurements the libraries were pooled in equimolar concentrations (libraries to be pooled are of the same concentration). The library pool(s) were finally quantified again with qPCR and optimal concentration of the library pool used to generate the clusters on the surface of a flowcell before sequencing using v2 sequencing methodology according to the manufacturer instructions (Illumina Inc.). Samples were sequenced on the Illumina NextSeq 500 system.

#### Analyses of RNA-seq and miRNA-seq Data

Following sequencing, intensity correction and base calling (into BCL files), FASTQ files were generated using the appropriate bcl2fastq software (Illumina Inc.) which includes quality scoring of each individual base in a read. We found that the vast majority of the data has a Q score greater than 30 (>99.9% correct), indicating that high quality data was obtained for all samples.

Principal Component Analysis was performed on miRNA samples using the base R function. To produce the hierarchically clustered heat map, the miRNA-seq data were initially filtered by removing any miRNAs that had a False Discovery Rate (FDR) of greater than 0.001 to improve readability of the heat map. All miRNAs with an FDR of < 0.001 were then hierarchically clustered using the built-in hierarchical clustering algorithm in the heatmap.2 function. The color-key for each heat map was created using predetermined break points to bin the TMM value into colors for each marker. The volcano plot was also obtained using the base R volcano plot function.

### *In situ* Hybridization

All samples were collected and quickly fixed in a modified Carnoy’s fixative overnight at 4°C. For the mouse embryonic samples, we fixed whole heads while postnatal day 3 and human fetal samples were fixed as whole eyes. A small hole was made with an 18 gauge needle at the corneal limbus to facilitate the fixation. After fixation, samples were dehydrated and embedded in paraffin as described elsewhere (Fairchild et al., 2018). Horizontal sections of whole embryo heads (mouse E13.5 and E16.5) and sagittal sections of whole eyes (mouse P3 and human 77–83 days) were prepared at 5 μm, collected onto SuperFrost slides, and air dried overnight at room temperature. Paraffin wax-embedded sections were baked for 45 min at 60°C, deparaffinized using xylene, rehydrated with ethanol (stepwise) and PBS, and treated with Proteinase K for 10 min at 37°C. A double digoxigenin (DIG)-labeled locked nucleic acid (LNA) ISH probe (miRCURY LNA Detection probe) was purchased from Exiqon/Qiagen. ISH was performed using the miRCURY LNA microRNA Detection FFPE microRNA ISH Optimization Kit 4 (Exiqon), which includes hybridization buffers and control probes (LNA scramble microRNA and LNA U6 snRNA control probe), according to manufacturer’s protocol. The following LNA miRNA probes were used for ISH: miR-15b-5p (Qiagen, Cat#YD00611174-BEG, 1:500), miR-30b-5p (YD00610927-BCG, miR-30b, 1:500), miR-93-5p (Qiagen, Cat#YD00611038, miR-93-5b, 1:300), miR-103-3p (Qiagen Cat#YD00612004, 1:500), miR-342-5p (Qiagen, Cat#YD00611489, 1:625), U6 (Qiagen, Cat#YD00699002-BEG, 1:500), scrambled (Qiagen Cat#YD00699004, 1:300). LNA probes were hybridized for 1 h at 55°C and rinsed with SSC buffer (stepwise from 5× to 0.2×). Sections were blocked in 2% sheep serum/1% bovine serum albumin/PBS-0.01% Tween for 30 min at room temperature. Detection was performed using an alkaline phosphatase conjugated anti-DIG secondary antibody (Roche) in 1% sheep serum/1% bovine serum albumin/PBS-0.05% Tween for 1 h at room temperature. Following rinsing in PBS-0.1% Tween, sections were incubated in developing solution of sodium chloride 0.1 M/tris pH 9.5 0.1M/magnesium chloride 10 mM/0.1% Tween-20 and NBT (nitroblue tetrazolium)/BCIP (5-bromo-4-chloro-3-indolyl phosphate) stock solution (Roche). After the reactions were deemed complete (1–4 days), sections were fixed with 4% paraformaldehyde and mounted for microscopy using Fluoromount-G (Southern Biotech).

### Immunofluorescence

Sections were prepared as described previously (Fairchild et al., 2018; Leger et al., 2019). Sections were then deparaffinized using xylene, rehydrated with ethanol (stepwise), rinsed with PBS-0.3% Triton X-100, and antigen retrieval was performed by treating the slides with 0.1 M sodium citrate. All sections were then blocked in 10% normal donkey serum/PBS-0.1% Triton X-100 in PBS for 1 h at room temperature and incubated in primary antibody in blocking solution overnight at 4°C. The following antibodies were used for immunofluorescence: goat anti-OTX2 (R&D Systems Cat#BAF1979), 1:500; rabbit anti-RBPMS (Phosphosolutions Cat#1832-RBPMS, 1:400, and anti-PCNA (Abcam Cat#ab18197, 1:500), and anti-PH3 (Thermo Fisher Cat#PA5-17869, 1:300). After primary antibody incubation, sections were rinsed in PBS and incubated with appropriate Alexa Fluor-conjugated secondary antibodies (Invitrogen, 1:300) in blocking solution for 1 h at 4°C. Cell nuclei were counterstained with DAPI. The sections were rinsed with PBS and mounted for microscopy using a Fluoromount-G (Southern Biotech).

### Hematoxylin and Eosin Staining

Samples were prepared as described previously (Fischer et al., 2008). Next, sections were deparaffinized using xylene, rehydrated with ethanol (stepwise) and water, and stained with hematoxylin and eosin, and dehydrated with ethanol (stepwise). The sections were then rinsed in xylene and mounted for microscopy using a Fluoromount-G (Southern Biotech).

### Microscopy

*In situ* hybridization were imaged using an Axio Imager M2 with ApoTome2 microscope system (Zeiss) using tile scan options (ZEN imaging software), and immunolabeling experiments were documented using a Fluoview FV3000 confocal microscope (Olympus). Images were processed using Fiji (ImageJ software), and figures were prepared in Adobe Photoshop 2000.

## Data Availability Statement

The datasets presented in this study can be found in online repositories. The names of the repository/repositories and accession number(s) can be found below: NCBI Gene Expression Omnibus, accession no: GSE168475

## Ethics Statement

The studies involving human participants were reviewed and approved by University of California Davis Institutional Review Board, University of California, Davis. Written informed consent for participation was not required for this study in accordance with the national legislation and the institutional requirements. The animal study was reviewed and approved by Institutional Animal Care and Use Committee; University of California, Davis.

## Author Contributions

EF, ML, AMi, SC, JW, and NS conducted experiments and/or analyses. AMo conducted sample collection, contributed to the study design, and revised the manuscript. AFT identified, selected, and monitored the pregnancies sonographically, and collected the specimens for analysis, contributed to the study design, and revised the manuscript. SS and ALT supervised and designed the study, conducted experiments, and wrote the manuscript. All authors contributed to the manuscript and approved the submitted version.

## Conflict of Interest

The authors declare that the research was conducted in the absence of any commercial or financial relationships that could be construed as a potential conflict of interest.

## Supplementary Material

Supplementary Figure 1 (related to Figure 1). **(A,B)** Heatmaps showing expression of cell-specific markers during *Macaca mulatta* retinal development. Comparisons between Temporal (T) and Nasal (N) data is shown at 50, 90, and 150 days gestational age. Expression of genes involved in panel **(A)** cell cycle and **(B)** Notch signaling pathway. All the data is expressed as CPMs.

Supplementary Figure 2 (related to Figure 2). MiENTURNET network analysis of differentially expressed miRNAs. **(A)** mRNA-miRNAs network of miRNAs enriched in the temporal samples **(B)** mRNA-miRNAs network of miRNAs enriched in the nasal samples. miRNAs are indicated as blue dots, while target genes are yellow dots. Relevant targets genes are indicated.

Supplementary Figure 3 (related to Figure 4). Close-ups of miRNA *in situ* hybridization in the mouse retina. Black arrows indicate regions with higher expression level while white arrows indicate areas that display lower levels of expression. Scale bar: 100 microns for the top panel (miR-15b-5p) and 200 microns for the other panels.

Supplementary Figure 4 Comparison between developmental timing in mouse, rhesus monkey, and human. The timing of key events during retinal histogenesis has been calculated using a prediction model previously published (translatingtime.org).

Supplementary Figure 5 (related to Figure 5). Hematoxylin and Eosin (H&E) staining and immunohistochemistry of human fetal retinas. **(A–A″)** H&E staining of human fetal retina at 77 days of gestation (H&E) staining. **(B–C′)** Immunohistochemistry using PH3 [green, white arrows in panels **(B,C)**], PCNA (red), RBPMS (gray) antibodies and counterstained with DAPI. **(D–E′)** OTX2 staining (green). The samples were also counterstained with DAPI (blue). **(F–G)** Quantification of the number of PH3+ **(F)** and OTX2+ cells **(G)** per 250 μm of retina in the temporal and nasal regions of the retina. L, lens; N, Nasal; T, Temporal; ON, optic nerve; NbL, neuroblastic layer; GCL, Ganglion cell layer; ^∗^ indicates the localization of the optic nerve head. Scale bars: 500 microns in panel **(A)**, 200 microns in panels **(A′–E″)**. Error bars indicate standard deviation.

Supplementary Figure 6 (related to Figure 5). miRNA *in situ* hybridization in the human retina. **(A–D)** miRNA expression in the human fetal retina at 70–82 days gestation. miR-93 **(A–A″)** and miR-30b **(B–B″)** expression in the developing human retina. **(A′,B′)** Inset pictures of the nasal retina. **(A″,B″)** Inset pictures of the temporal retina at the foveal anlage. Scale bars: 500 microns in panels **(A,B)**, and 200 microns in panels **(A′, A″, B′,B″)**.

Supplementary Figure 7 (related to Figure 5). Close-ups of miRNA *in situ* hybridization in the human fetal retina. miR-15b expression in the human fetal retina at 95 days gestation. miR-15b is enriched in the temporal progenitors (black arrows). Immunolabeling experiments using OTX2 (blue), PAX6 (green) and PCNA (red) using consecutive sections. Scale bar: 50 microns.

Supplementary Table 1 (related to Figure 1). mRNA-sequencing results of *Macaca mulatta* retinas.

Supplementary Table 2 (related to Figure 2). miRNA-sequencing results of *Macaca mulatta* retinas

Supplementary Table 3 (related to Supplementary Figure 2). MIRTURNET target enrichment for the temporal network.

Supplementary Table 4 (related to Supplementary Figure 2). MIRTURNET target enrichment for the nasal network.
