# Supplementary figures and images for: MicroRNA Signatures of the Developing Primate Fovea
Source: Front Cell Dev Biol. 2021 Apr 8;9:654385. doi: 10.3389/fcell.2021.654385 (PMC8060505; doi:10.3389/fcell.2021.654385)

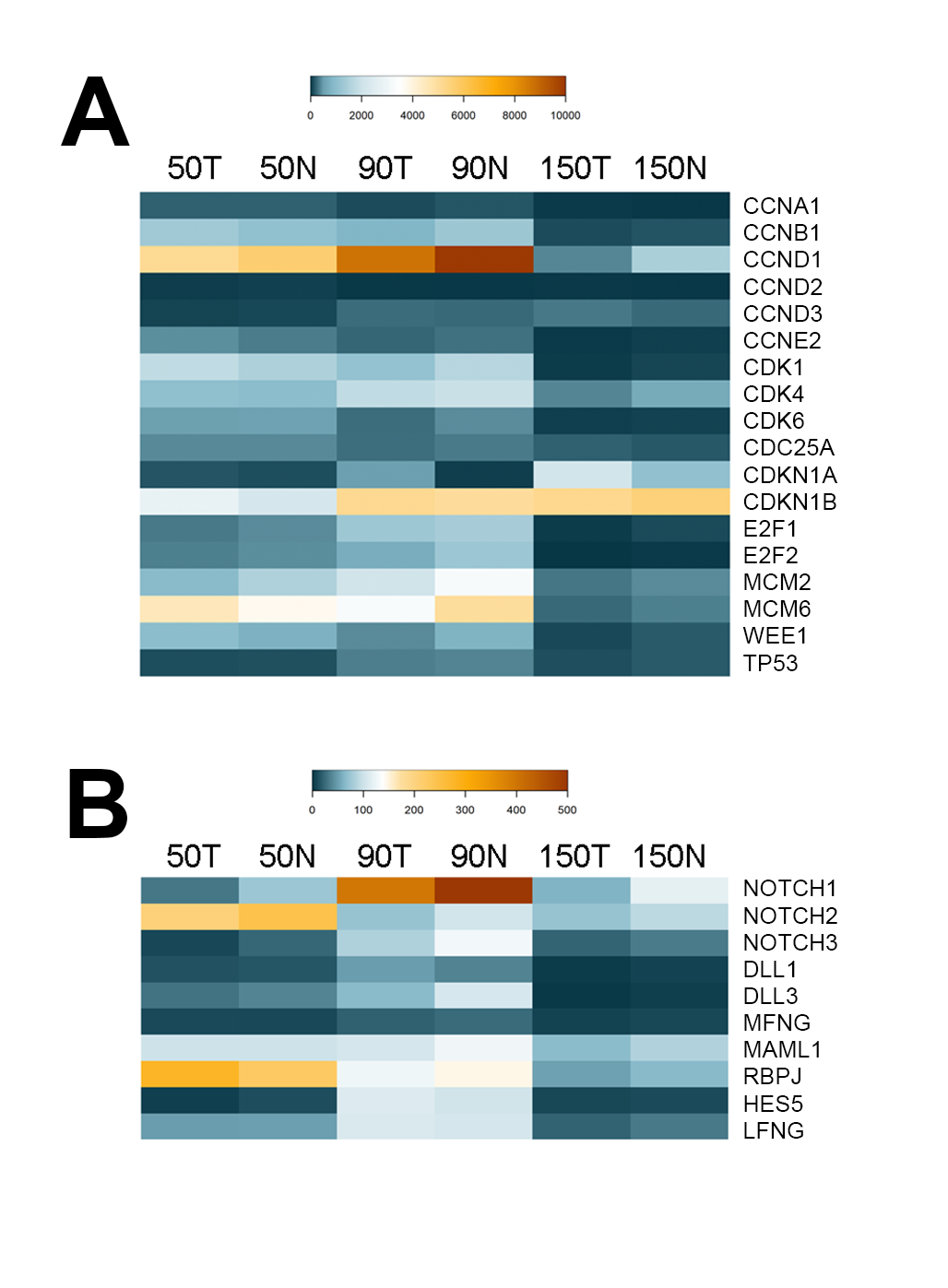

Supplement: Supplementary Figure 1 — (related to Figure 1). (A,B) Heatmaps showing expression of cell-specific markers during Macaca mulatta retinal development. Comparisons between Temporal (T) and Nasal (N) data is shown at 50, 90, and 150 days gestational age. Expression of genes involved in panel (A) cell cycle and (B) Notch signaling pathway. All the data is expressed as CPMs. [file Image_1.TIF]

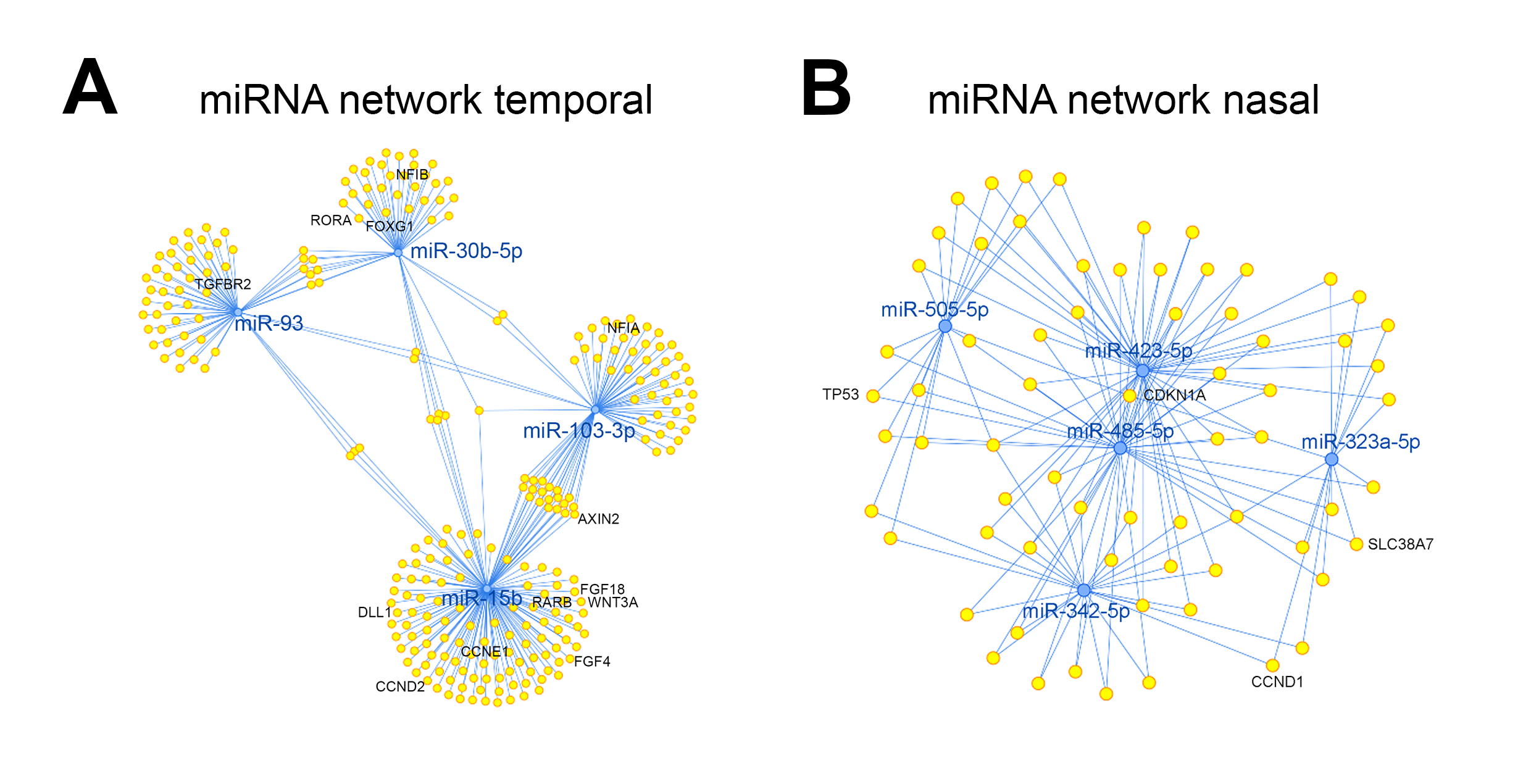

Supplement: Supplementary Figure 2 — (related to Figure 2). MiENTURNET network analysis of differentially expressed miRNAs. (A) mRNA-miRNAs network of miRNAs enriched in the temporal samples (B) mRNA-miRNAs network of miRNAs enriched in the nasal samples. miRNAs are indicated as blue dots, while target genes are yellow dots. Relevant targets genes are indicated. [file Image_2.TIF]

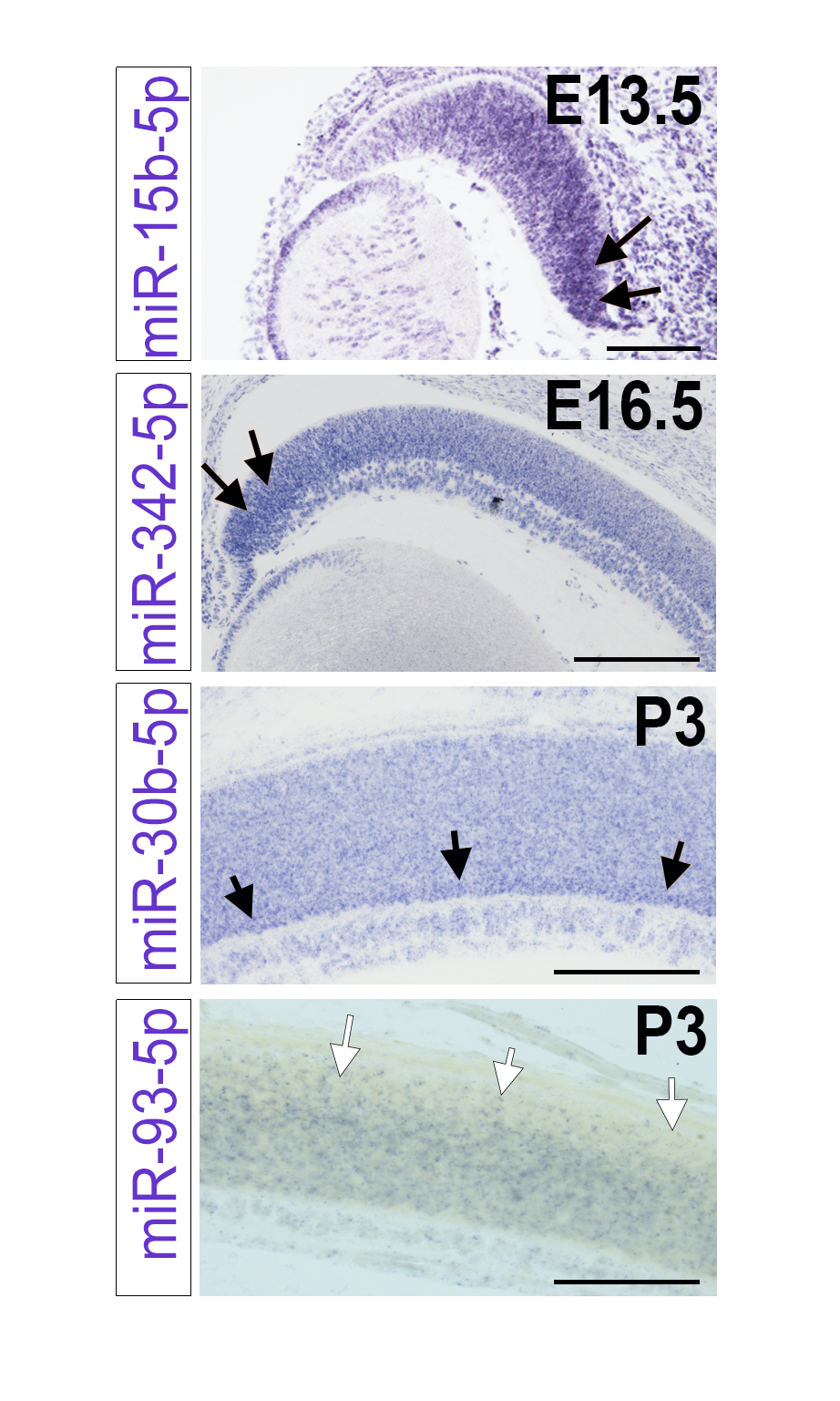

Supplement: Supplementary Figure 3 — (related to Figure 4). Close-ups of miRNA in situ hybridization in the mouse retina. Black arrows indicate regions with higher expression level while white arrows indicate areas that display lower levels of expression. Scale bar: 100 microns for the top panel (miR-15b-5p) and 200 microns for the other panels. [file Image_3.TIF]

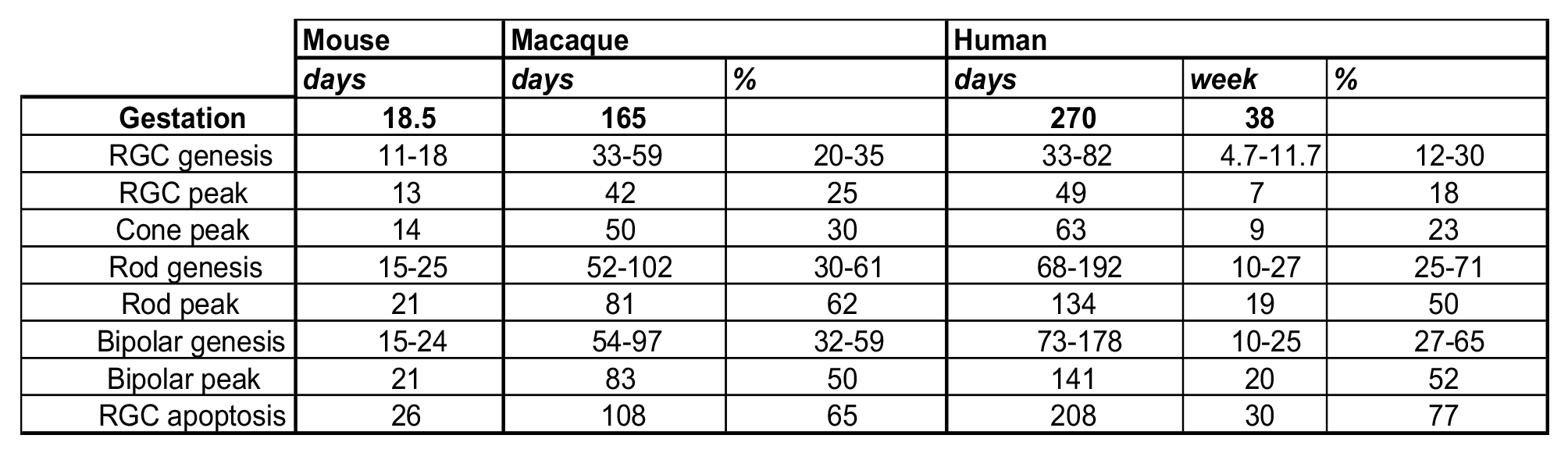

Supplement: Supplementary Figure 4 — Comparison between developmental timing in mouse, rhesus monkey, and human. The timing of key events during retinal histogenesis has been calculated using a prediction model previously published (translatingtime.org). [file Image_4.TIF]

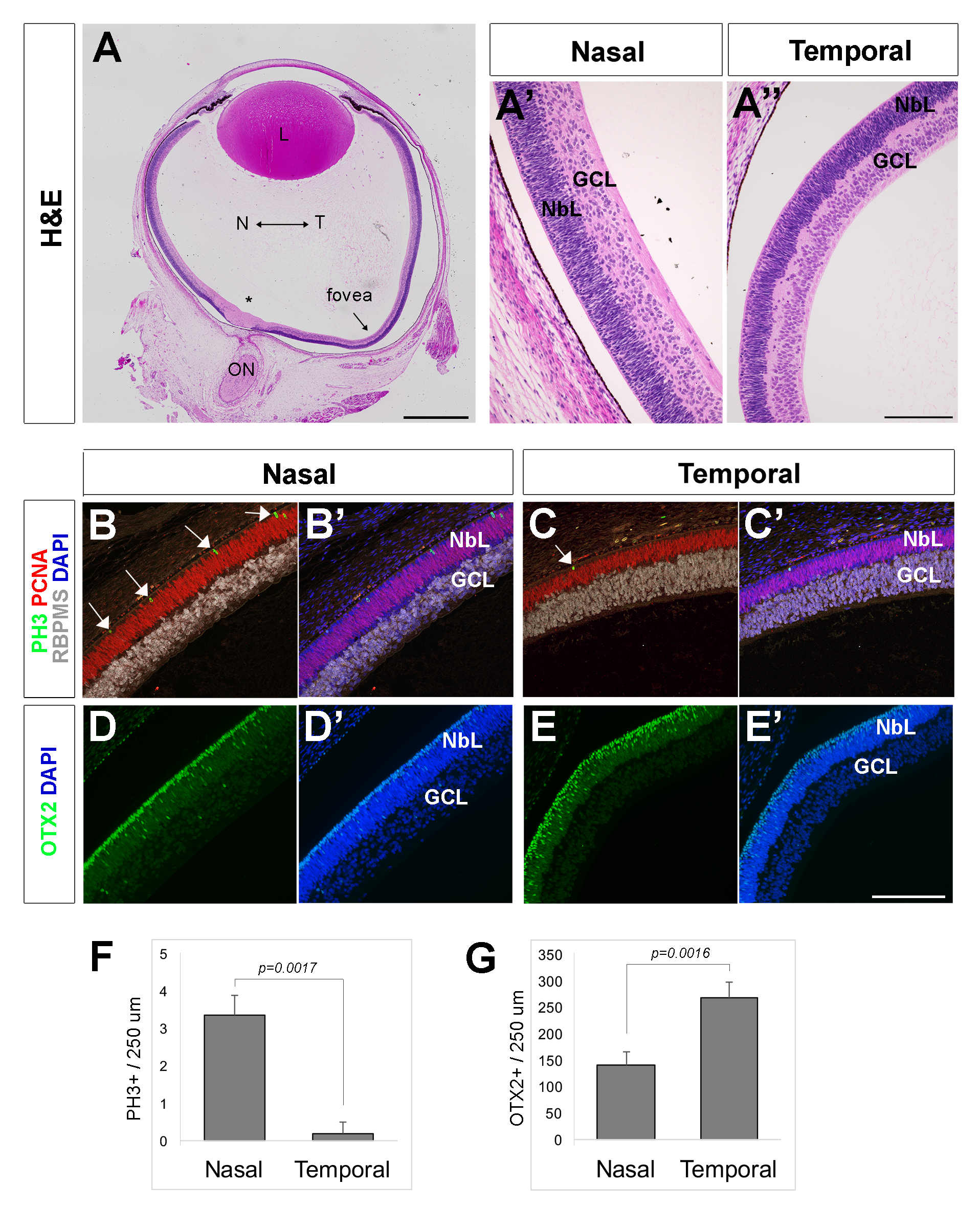

Supplement: Supplementary Figure 5 — (related to Figure 5). Hematoxylin and Eosin (H&E) staining and immunohistochemistry of human fetal retinas. (A–A″) H&E staining of human fetal retina at 77 days of gestation (H&E) staining. (B–C′) Immunohistochemistry using PH3 [green, white arrows in panels (B,C)], PCNA (red), RBPMS (gray) antibodies and counterstained with DAPI. (D–E′) OTX2 staining (green). The samples were also counterstained with DAPI (blue). (F–G) Quantification of the number of PH3+ (F) and OTX2+ cells (G) per 250 μm of retina in the temporal and nasal regions of the retina. L, lens; N, Nasal; T, Temporal; ON, optic nerve; NbL, neuroblastic layer; GCL, Ganglion cell layer; ∗ indicates the localization of the optic nerve head. Scale bars: 500 microns in panel (A), 200 microns in panels (A′–E″). Error bars indicate standard deviation. [file Image_5.TIF]

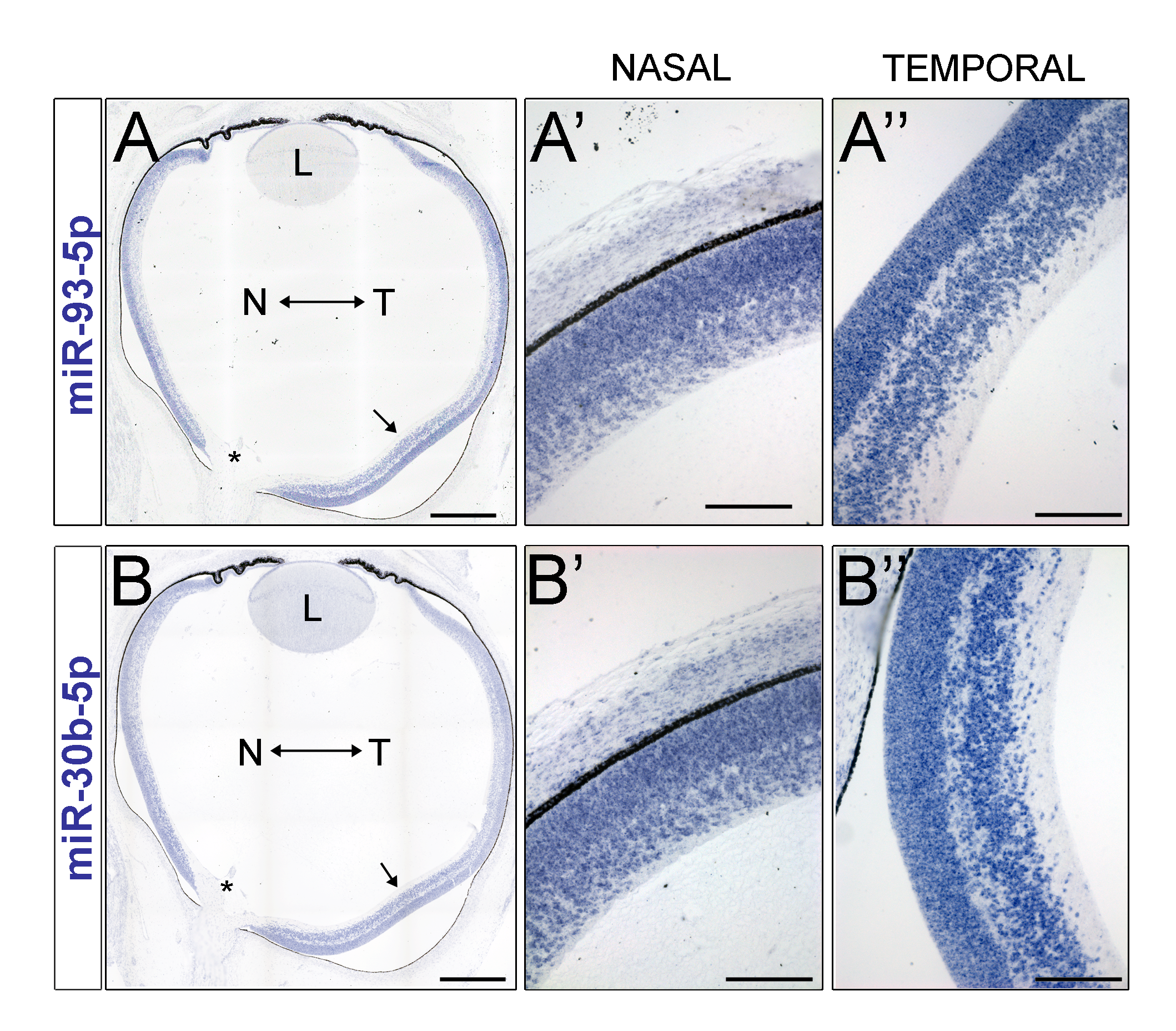

Supplement: Supplementary Figure 6 — (related to Figure 5). miRNA in situ hybridization in the human retina. (A–D) miRNA expression in the human fetal retina at 70–82 days gestation. miR-93 (A–A″) and miR-30b (B–B″) expression in the developing human retina. (A′,B′) Inset pictures of the nasal retina. (A″,B″) Inset pictures of the temporal retina at the foveal anlage. Scale bars: 500 microns in panels (A,B), and 200 microns in panels (A′, A″, B′,B″). [file Image_6.TIF]

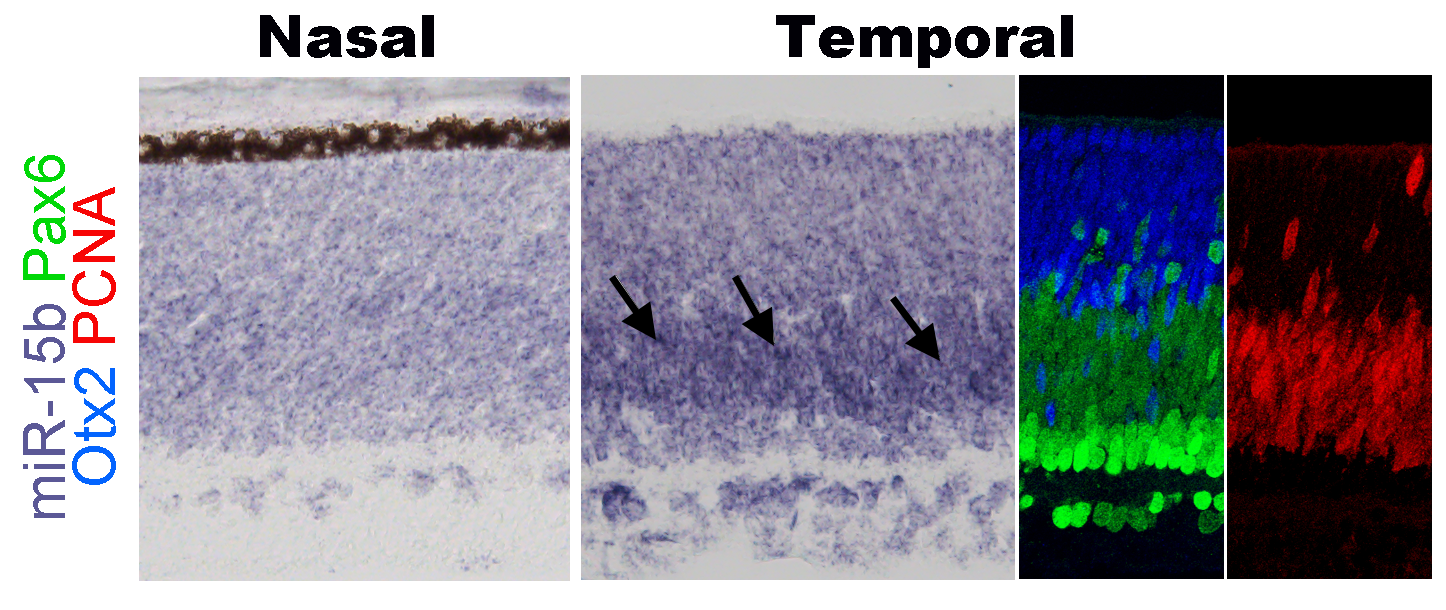

Supplement: Supplementary Figure 7 — (related to Figure 5). Close-ups of miRNA in situ hybridization in the human fetal retina. miR-15b expression in the human fetal retina at 95 days gestation. miR-15b is enriched in the temporal progenitors (black arrows). Immunolabeling experiments using OTX2 (blue), PAX6 (green) and PCNA (red) using consecutive sections. Scale bar: 50 microns. [file Image_7.TIF]
